# Regulation of mTOR, Metabolic Fitness, and Effector Functions by Cytokines in Natural Killer Cells

**DOI:** 10.3390/cancers9100132

**Published:** 2017-09-28

**Authors:** Sébastien Viel, Laurie Besson, Marie Marotel, Thierry Walzer, Antoine Marçais

**Affiliations:** 1 Centre International de recherche en Infectiologie, CIRI, Inserm, U1111, Université Claude Bernard Lyon 1, CNRS, UMR5308, École Normale Supérieure de Lyon, University of Lyon, 69007 Lyon, France; laurie.besson@inserm.fr (L.B.); marie.marotel@inserm.fr (M.M.); thierry.walzer@inserm.fr (T.W.); 2 Laboratoire d’Immunologie, Hospices Civils de Lyon, Centre Hospitalier Lyon Sud, 69310 Pierre-Bénite, France

**Keywords:** NK cells, metabolism, mTOR, IL-15, TGF-β

## Abstract

The control of cellular metabolism is now recognized as key to regulate functional properties of immune effectors such as T or Natural Killer (NK) cells. During persistent infections or in the tumor microenvironment, multiple metabolic changes have been highlighted in T cells that contribute to their dysfunctional state or exhaustion. NK cells may also undergo major phenotypic and functional modifications when infiltrating tumors that could be linked to metabolic alterations. The mammalian target of rapamycin (mTOR) kinase is a central regulator of cellular metabolism. mTOR integrates various extrinsic growth or immune signals and modulates metabolic pathways to fulfill cellular bioenergetics needs. mTOR also regulates transcription and translation thereby adapting cellular pathways to the growth or activation signals that are received. Here, we review the role and regulation of mTOR in NK cells, with a special focus on cytokines that target mTOR such as IL-15 and TGF-β. We also discuss how NK cell metabolic activity could be enhanced or modulated to improve their effector anti-tumor functions in clinical settings.

## 1. Introduction

Natural killer (NK) cells are Innate Lymphoid Cells (ILCs) able to kill abnormal cells recognized as targets and to produce large amounts of IFN-γ and other cytokines and chemokines upon activation [1]. This allows them to take part in the immuno-surveillance of cancers [1]. Indeed, they express a restricted set of receptors allowing them to discriminate normal from abnormal, pathogen-infected or tumor cells. NK cell receptors have activating or inhibitory properties upon engagement by molecules displayed at the surface of target cells. The balance between activating and inhibitory signals controls immediate effector functions: cytotoxicity and IFN-γ secretion. As previously reviewed, the triggering of these effector functions is metabolically demanding and requires energy, especially when triggering NK cell receptors or under limited exposure to IL-15 [2]. However, NK cell metabolism may be different than that of T cells, as unlike these cells, they do not need to proliferate to display effector functions upon activation.

Multiple articles have demonstrated the capacity of NK cells to limit tumor growth in vivo in mouse models of melanoma, myeloma, lymphoma, or other cancer cell types, as previously reviewed [3]. Yet, in most cases, NK cell anti-tumor activity is overwhelmed when large numbers of tumor cells are injected [4]. Tumor growth is also associated with a progressive impairment of NK cell function, manifested by reduced expression of activating receptors and decreased effector functions [5]. NK cell exhaustion can also be associated in some cases with up regulation of inhibitory receptors such as PD-1 [6]. The latter observation is more frequently made for exhausted T cells in various settings of cancer or chronic infection. T cell exhaustion is also linked with a progressive impairment of bioenergetics metabolism, both glycolytic and respiration-associated. For example, during chronic Lymphocytic choriomeningitis virus (LCMV) infection in mice or during the course of Hepatitis B virus (HBV) chronic infection in human, in parallel with the development of dysfunction, virus-specific CD8+ T cells are unable to match the bioenergetics of effector T cells generated during acute infection [7,8]. Suppression of T cell bioenergetics involved restricted glucose uptake and use, despite persisting mechanistic target of rapamycin (mTOR) signaling. Mechanistically, PD-1 regulated early glycolytic and mitochondrial alterations in part by repressing the transcriptional coactivator PGC-1α [9]. In another study, it was reported that T cells infiltrating tumors show decreases in mitochondrial function and mass, leading to loss of oxidative respiration. T cell mitochondrial biogenesis was repressed via Akt-mediated inhibition of PGC-1α [10]. T cell dysfunction was also linked to an increased expression of a gene module involved in zinc metabolism, suggesting that the adaptive gain of metabolic pathways in the tumor environment may also contribute to their altered function [11]. Importantly, improving bioenergetics by overexpression of PGC-1α may enhance function in exhausted T cells, both in cancer and infection settings. Whether this is also the case for NK cells requires further investigation but multiple recent articles reported that several cytokines may control NK cell metabolism by regulating the activity of the mTOR kinase. Here, we review the corresponding literature and discuss how metabolic activity could be reinvigorated in NK cells to enhance their anti-tumor activity.

## 2. IL-15 Activates mTOR in NK Cells and Boosts Cellular Metabolism

At steady state, mouse as well as human NK cells are moderately cytotoxic, they also present a low basal bioenergetics metabolism, characterized by low levels of glycolysis and oxidative phosphorylation (OxPhos) as measured by the SeaHorse technology [12,13,14,15] (Table 1).

This correlates with poor expression of nutrient transporters and glucose uptake, a state also reflected by limited NK cell size. In this resting state, basal metabolic activity, and in particular OxPhos, is necessary for IFN-γ secretion triggered by NK cell stimulation through the activating receptors NKRP1A, NKp46, and Ly49D in mice [13] or for IFN-γ secretion and degranulation induced by cytokines in human [15]. Similarly, deficiency in the metabolic checkpoint kinase mTOR leads to decreased metabolic fitness of resting NK cell and lower responses to activating receptor stimulation.

In vivo, NK cell priming following injection of the dsRNA mimetic poly(I:C), a potent inducer of IL-15, strongly increases NK cell metabolic activity [12,14]. At the molecular level, this correlates with a strong activation of the two complexes containing mTOR: mTORC1 and C2 [12,14]. This evolutionarily conserved serine/threonine kinase integrates various extracellular cues: metabolite and growth factors but also antigenic and inflammatory signals and controls in return numerous metabolic pathways. In agreement with the known role of IL-15 in NK cell priming [18], antibody mediated blocking of its receptor identified this cytokine as the chief but non-exclusive source of extracellular signaling that leads to mTOR hyperactivation [12]. Moreover, as it was previously demonstrated for priming of effector functions, in vitro IL-15 treatment mimicked poly(I:C) treatment and activated NK cell metabolism [12,13,14]. This was characterized by a shift in the balance from OxPhos to glycolysis, a phenomenon previously described in activated T cells. This increase in metabolic activity relies on mTOR, with mTORC1 activity controlling the expression of several genes necessary for glycolysis activation [14]. mTOR activity is tightly regulated during NK cell maturation. In mice, phosphorylation of mTOR itself or its targets decreased in a coordinated fashion upon maturation [12]. In the same way, human immature (CD56^bright^) NK cells display higher response to IL-15 stimulation in terms of S6 phosphorylation in vitro or in terms of proliferation and functionality after IL-15 treatment in vivo [19].

Importantly, glycolytic metabolism increase is necessary for the priming of NK cell effector function as judged by lower intracellular Granzyme B content upon glycolysis inhibition [14]. Accordingly, mTORC1 inhibition or acute mTOR deletion also prevents increases in cytolytic and IFN-γ secretion potentials in NK cells stimulated with IL-15 [12,14]. While not investigated directly, mTORC2 activity probably also plays a role since mTOR deficiency or mTORC1/2 inhibitors have more profound effect than mTORC1 inhibitors [12]. This point requires formal genetic testing, as no mTORC2 specific inhibitors are available yet.

Once the priming is effective, NK cells become independent of extracellular metabolic cues. Indeed, Keppel et al. demonstrate that NK cell priming leads to OxPhos-independent capacity to secrete IFN-γ in response to NK receptor stimulation [13]. More surprisingly, they also present data suggesting that NK cells become to some extent independent of extracellular glucose and therefore of glycolysis [13]. If confirmed, this would endow primed NK cells with enhanced functionality in nutrient deprived locations such as tumor microenvironment [17,20]. In the same line of thought, the IL-12/18 cytokine cocktail is able to induce NK cell production of IFN-γ even in the presence of metabolic inhibitors or in the absence of mTOR [12,13]. In both cases, it appears as if innate immune signals activating NK cells are able to supersede the metabolic requirement of these cells.

As it is now described for an increasing number of immune cell subsets, these studies unmask the profound relationship existing between cell metabolism and effector functions in NK cells. In the resting state, NK cell effector functions triggered by activating receptors are highly dependent on metabolism, specifically OxPhos. Similarly, IL-15 priming also relies on mTOR-dependent metabolism. The molecular mechanisms linking higher metabolic activity and enhanced effector functions are still obscure. Trivial reasons like better macromolecular synthesis capacities could obviously account for more efficient effector molecules production and secretion. In this respect, a recent study investigating the relative effect of IL-2 and IL-15 in in vitro treatment described a better persistence of IL-15 effects on NK cells anti-tumor functions upon cytokine withdrawal [21]. This was associated with the activation of the mTOR pathway and better translation of a number of transcripts as measured by polysome association. It seems that IL-15 has a superior priming activity associated with a stronger capacity to reprogram cell metabolism. Inhibition of glycolysis is also linked to the up regulation of immune regulatory receptors such as PD-1 in T cells [22]. In addition, more direct relations between metabolism and effector functions could exist as recently described for the moonlighting of Glyceraldehyde 3-phosphate dehydrogenase (GAPDH) in T cells [23]. GAPDH associates with the 3′ untranslated region of IFN-γ mRNA thereby regulating its translation, in relation with glycolysis levels. Moreover, some metabolites can act as signaling molecules. For example, succinate, which abundance is correlated with TCA cycle flux within the cell, may have a key role in inflammation through stabilization of HIF-1α and inflammatory cytokine transcription [24]. Similarly, phosphoenolpyruvate is involved in the control of Calcium flux [25] and mitochondrial reactive oxygen species (ROS) regulate T cell activation, so that disruption of mitochondrial electron transports impairs T cell activation [26]. Further work is needed to fully understand how mTOR regulates T/NK cell reactivity and effector functions. In particular, comprehensive studies such as proteomics, phospho-proteomics, and analysis of metabolic fluxes are necessary to address the full range of mTOR activities on NK cell metabolic pathways and beyond.

## 3. TGF-β Represses the mTOR Pathway in NK Cells

TGF-β is a cytokine with pleiotropic functions in physiological and pathological processes [27]. Active TGF-β binds to its receptor (TGF-βR), which is composed of two TGFbRI and two TGFbRII chains. TGF-β induces the phosphorylation of the transcription factors Smad2 and Smad3. This complex then binds to Smad4 (or other co-Smad factors like TIF1γ) and represses or induces the expression of target genes. In addition to this Smad-dependent pathway, TGF-β activates other “non-canonical” pathways such as MAPK, PI3K-Akt, or TRAF6 depending on the cell type and context [28]. In the immune system, TGF-β is considered as one of the most powerful immunosuppressive cytokines [29]. It is known to suppress the activity of adaptive and innate cells like NK cells [30,31]. Indeed, TGF-β inhibits IL-2 induced proliferation, cytotoxicity and IFN-γ secretion of NK cells [32,33]. Moreover, a transgenic expression of a dominant-negative form of TGF-βR in CD11c+ cells (including NK and dendritic cells) leads to an important NK cell expansion and an increase in their functional potential [34]. Several tumors like melanoma [35] or multiple myeloma [36] are known to produce TGF-β, which promotes cancer escape. The current understanding of the underlying mechanism is that TGF-β inhibits the expression of several NK cell activation receptors involved in the recognition of tumor cells, like NKp30 and NKG2D [37,38]. On the other hand, the deletion of TGF-βR in T cells leads to their differentiation in NK-cell like cells suggesting that TGF-β is a potent inhibitor of NK cell receptor expression [39]. Finally, a recent study has shown that TGF-β expression in the context of tumors may induce the conversion of effector NK cells into ILC1-like cells co-expressing CD49a and CD49b, whose expression of TNF-α may promote tumor growth [40]. Surprisingly, this phenomenon may be impeded by SMAD4 [41].

For a long time, the mechanism by which TGF-β inhibits NK cell function has remained unclear. It has been proposed that Smads proteins, signal transducers of TGF-β signaling, repress the expression of Tbx21, the gene coding T-bet, thereby inhibiting IFN-γ production by NK cells [42,43]. The fact that CD122, the β chain of IL-2/15 receptor, is a target of T-bet also explains that TGF-β indirectly decreases overall IL-15 signaling in NK cells. In a recent work, trying to understand the mechanism by which TGF-β inhibits NK cells function, we found that the absence of TGF-β signaling does not alter NK cell development, but strongly increases anti-tumor response [16] in line with the role of this cytokine in tumor escape. We observed that TGF-β inhibits mTOR signaling and has similar effects as those induced by the mTORC1 specific inhibitor rapamycin on NK cell metabolism. Indeed, TGF-β induces a decrease of the expression of metabolic receptors (CD98, CD71), of the cell size, of the ability to incorporate glucose and also inhibits glycolysis and oxidative phosphorylation [16].

Previous studies already showed that TGF-β and rapamycin present similar effects on other cell types. For example, TGF-β can be replaced by rapamycin to induce the differentiation of naïve T cells into Tregs cells [44]. Moreover, TGF-β and rapamycin both inhibit anti-CD3/CD28 induced proliferation in vivo [45]. Rapamycin and its derivatives are commonly used to prevent graft rejection and to prevent/treat graft vs. host diseases. The main mechanism of action of these drugs are inhibition of T cell proliferation, inhibition of dendritic cell maturation and induction of the expression of Foxp3, the master regulator of Tregs development [46]. Similarities between the effects of TGF-β and rapamycin are not limited to metabolism inhibition. In mice, both molecules inhibit NK cell proliferation, cytotoxicity and also the expression of numerous NK cell phenotypic markers [16]. In human NK cells, TGF-β and rapamycin also inhibit NK cell degranulation and their ability to secrete cytokines as far as granzyme B and perforin expression [16]. However, there were also some discrepancies as TGF-β induces the expression of TRAIL (Tumor-necrosis-factor related apoptosis inducing ligand) whereas rapamycin inhibits it. Conversely, rapamycin, but not TGF-β induces the expression of CD62L [16]. Recent studies already pointed out the induction of CD62L by rapamycin on CD8 T Cells [47].

Inhibition of the mTOR pathway by TGF-β was also supported genetically. NK cells from mice expressing a constitutively activated form of the TGF-β receptor (NK-tgfbr1CA) display a phenotype very close to that of mTOR deficient NK cells [12,16]. In both genetic models, there was an important reduction in the number of NK cells in the spleen consequent to a strong disruption of the NK cell maturation program. In both models, NK cells also expressed decreased levels of various proteins known to be regulated by mTOR (T-bet, Granzyme B, CD122, KLRG1). However, as for in vitro experiments, the expression of other markers (2B4, NKG2D, CD11c) was discordant between both genotypes, reinforcing the idea that the effects of TGF-β are not limited to inhibition of the mTOR pathway. Both models are also characterized by a reduction in steady-state NK cell proliferation, and a reduction of NK cell response after a challenge with MHC class I deficient cells. Concerning mTOR activity, it was observed that after a short stimulation with IL-15, NK-tgfbr1CA displayed a reduction of the expression of pS6, whereas pSTAT5 was not modified.

**Figure 1:**
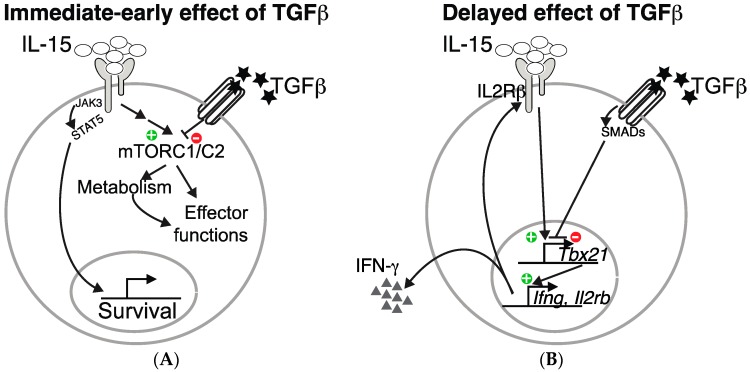
TGF-β inhibits IL-15-driven natural killer (NK) cell effector functions at two distinct levels. TGF-β inhibits IL-15 induced mTORC1/2 activation and the subsequent metabolic increase as well as effect on effector functions (**A**). In addition, it also inhibits the transcription of Tbx21, an IL-15 target, thus resulting in decreased IFN-γ production and IL2Rβ level (**B**).

In addition to these immediate-early effects of TGF-β on mTOR signaling, long lasting effects are also probably induced. Indeed, as already mentioned, TGF-β inhibits T-bet expression and consequently, the expression of one of its target: CD122, the β chain of the IL-15 receptor, thus contributing to decreased overall IL-15 signaling in NK cells. These two levels of control of NK cell functions by TGF-β are summarized in the Figure 1.

The molecular details responsible for the rapid inhibition of the mTOR pathway by TGF-β signaling are unknown. T-bet inhibition by Smad3 was proposed to explain the inhibition of IFN-γ production in mice and human NK cells [42,43]. However, in the absence of T-bet, TGF-β is still able to inhibit the phosphorylation of S6 at early time points and NK cell proliferation at later times [16]. The protein FKBP12 (FK Binding Protein 12) constitutes a potential link between TGF-β signaling and mTOR. Indeed, in order to inhibit mTOR activity, Rapamycin binds to FKBP12 [48]. In addition, FKBP12 constitutively binds to TGFbRI [49] and is only released after TGFbRI engagement. Whether natural ligands of FKBP12, able to regulate mTOR activity, exist is unknown. Besides, Treg development, which depends on both TGF-β and mTOR inhibition, is strongly skewed in mice lacking FKBP12 [50]. Moreover, in transplanted patients, treatment with FK506, another chemical FKBP12 ligand, decreases the percentage of Tregs in the periphery [51]. Our unpublished data have shown that some of the inhibitory effects of TGF-β on mTOR can be blocked by the addition of FK506 suggesting that at least in part, some of the effects of the TGF-β are mediated by FKBPs. Another possible mechanism to explain the effects of TGF-β on mTOR is an interaction with the phosphatase PP2A (Protein phosphatase 2A). This protein is a serine/threonine phosphatase highly conserved that regulates key cellular process such as cell cycle, apoptosis, migration, and cellular metabolism [52]. In Treg cells, PP2A inhibits mTORC1 and the ablation of PP2A leads to autoimmunity, a phenotype reminiscent of what is observed in case of ablation of TGFbRII [53]. Whether this phosphatase plays a role in the regulation of NK cell functions by TGF-β remains to be explored.

## 4. Strategies to Boost NK Cell Metabolism

Approaches to manipulate NK cell effector functions are being investigated in the cancer field in preclinical models or in clinical trials using a highly diverse range of approaches [54]. These include blocking of the inhibitory receptors to tilt the balance of signal towards activation [55,56] or redirecting NK cell specificity toward carefully chosen targets using bispecific killer engagers (BiKEs) [57] or chimeric antigen receptors (CAR) [58,59]. In addition, given that NK cell effector functions are greatly enhanced by cytokines, another immunotherapeutic approach consists in boosting NK cell effector functions globally using cytokines. This strategy is deemed to restore effector functions in the exhausted NK cell population found in situation of advanced stages of tumorigenesis [4,5,60]. Several cytokines or cytokine cocktails have been tested in preclinical models or in clinical trials to date such as IL-15 or IL-15 derived molecules [61,62], IL-2 [63], IL-21 [64], or an IL12/18/15 cocktail [4,65,66]. Combination of different strategies, such as the development of Trispecific Killer Engager (TriKE), are also being considered [67].

As discussed here, therapies should aim at inducing optimal mTOR activity and bioenergetics metabolism in NK cells. In this context, IL-2, IL-15, and derivatives appear as obvious candidates. Unlike IL-2, IL-15 does not induce Treg expansion and seems therefore more appropriate. IL-15 therapy would benefit from the inhibition of negative feedback regulators such as the cytokine-inducible SH2-containing protein (CIS, encoded by Cish). Cish is rapidly induced in NK cells in response to IL-15, and deletion of Cish renders NK cells hypersensitive to IL-15, by increasing JAK/STAT signaling and enhances cytotoxicity toward tumors [68]. We previously showed that IL-18 could also induce mTORC1 activity [12] and this may account for the synergy between IL-18 and IL-12/IL-15 in the induction of optimal NK cell effector functions in adoptive transfer experiments [65]. Blockade of inhibitory cytokines such as TGF-β is also desired and could be combined with IL-2/15. Multiple strategies have been designed to block TGF-β, TGF-β activation, or signaling and are currently under preclinical development or clinical testing [69]. This includes reagents that (i) directly inhibit TGF-β synthesis by antisense molecules; (ii) block TGF-β and its interaction with its receptor using monoclonal antibodies or soluble TGF-β decoy receptors; or (iii) inhibit TGF-β signaling with kinase inhibitors or aptamers that interfere with the function of the downstream Smad signaling proteins. For instance, a previous study reported that the combined delivery of TGF-β inhibitor and IL-2 by nanoscale liposomal polymeric gels enhanced tumor immunotherapy by promoting NK and CD8 T cell functions [70]. Going a step further, a recent preclinical study described the association of IL-15 with a TGF-β trapping subunit in a fusion molecule [71]. In addition, some trials are already ongoing to test the combined effect of anti-PD1 antibodies with TGF-β blockers [69].

Blockade of PD-1 and CTLA-4 may increase the metabolic fitness of effector T cells by enhancing the activity of the PI3K/mTOR pathway and increasing cMyc expression, which then stimulates aerobic glycolysis and anabolic metabolism [72]. As discussed above, multiple NK cell receptors or other receptors co-expressed by T and NK cells are being targeted with agonistic or blocking antibodies. However, a recent study reported negative results upon administration of IPH2101, a KIRD blocking antibody, to myeloma patients [73], suggesting that combination of several strategies might be necessary to both induce NK cell effector functions while preserving their responsiveness. In this respect, monitoring of mTOR activity should be performed as a correlate of their metabolic fitness (see below). It will also be important to carefully determine the interplay between NK cell receptors and mTOR activity using preclinical models. In T cells, the TCR is indeed a major regulator of mTOR activity and metabolic pathways [74]. Similarly, in NK cells, we recently discovered that in the minutes following stimulation, triggering of activating receptors increases mTORC1/2 activity [75]. Furthermore, we describe that optimal mTOR activity maintains an adequate metabolic fitness and is required for maximal calcium flux and integrin activation following stimulation, thus linking mTOR activity to NK cell responsiveness. Physiologically, we report that this control of basal mTOR activity is linked with a process known as education whereby cell reactivity is adjusted as a function of the environment [75]. Interestingly, if the activating signal is maintained over several hours, we observed a depression of the activity of both mTOR-containing complexes concomitant with a loss of responsiveness toward subsequent stimulation [75]. Whether other recently discovered NK cell subsets (i.e., adaptive vs. canonical) also present specific regulation of mTOR activity is currently unknown but likely, since inhibition of mTOR activity after a viral challenge improves the generation of memory NK cells [76]. With respect to NK cell exhaustion, novel immune checkpoints, such as Tim3 or TIGIT whose expression is induced on tumor infiltrating NK cells [41,65], might regulate mTOR activity in NK cells as previously shown for PD-1 in T cells and thereby control cell reactivity. Further work is undoubtedly required to clarify these points.

More direct pharmacological activators of mTOR could also be envisaged. However, very few have been described and none of them have been tested in preclinical models of anti-tumor immune responses. Obviously, such molecules would ideally only target immune cells. Profiling experiments might lead to the discovery of immune-specific mTOR partners that would represent good candidates. The pharmacological arsenal to inhibit mTOR is by contrast very rich and diverse [77]. Several of these molecules are currently used for cancer treatment. Like many chemotherapies that non-specifically target cell proliferation, these mTOR inhibitors are expected to impede NK cell effector functions, even though this point requires formal testing.

Interestingly, several small molecules have been shown to enhance NK cell activity. This is the case of lenalidomide and other immuno-modulatory molecules of the iMID family such as thalidomide or pomalidomide. The proposed mechanism of action includes both direct effects [78] by lowering NK cell activation threshold and indirect effects via the induction of IL-2 production by CD4 T cells. The latter may promote mTOR activity in NK cells. Very little evidence of an NK cell stimulatory effect of iMIDs in patients is available. Yet, several clinical trials are undergoing that combine antibodies against NK cell receptors with lenalidomide, on the basis of preclinical results showing the efficacy of such combinations [79]. Tyrosine kinase inhibitors such as Imatinib mesylate have also been shown to enhance NK cell effector potential through unknown mechanisms [80]. More recently, it was found that the combination of MEK inhibitors paradoxically synergized with anti-PD1 antibodies to promote infiltration of tumors with effector T cells [81]. The molecular mechanism of tyrosine kinase inhibitors and the range of their actions on the immune system remains to be clarified, but an attractive possibility would be that such inhibitors could block NK or T cell exhaustion by preventing overt stimulation on the tumor bed. As T cell exhaustion is clearly linked with reduced mTOR activity and bioenergetics metabolism, kinase inhibitors might prevent the induction of negative feedback loops inhibiting mTOR activity.

## 5. Concluding Remarks

Here we reviewed how IL-15 controls NK cell metabolism and how TGF-β inhibits NK cell metabolism and function by blocking the mTOR pathway. The effects of IL-15 and TGF-β on NK cells were observed both in mice and humans, which suggests that this mechanism is conserved upon evolution. Numerous aspects still need to be deciphered. First of all, the relative contribution of mTORC1 and mTORC2 as well as the downstream effectors of these complexes are unknown. How the metabolic pathways enhance effector functions also remains mostly enigmatic. The identification of metabolites that positively regulate NK cell functions may pave the way for the development of novel therapies to boost anti-tumor responses. Moreover, NK cell exhaustion remains poorly defined, in spite of recent advances [82]. A better understanding of the exhaustion mechanism may also lead to novel strategies to improve the function of endogenous or transplanted NK cells. Finally, future works should concentrate on the impact of chemotherapeutics on NK cell activity for selection of the optimal clinical conditions for NK cell-based therapies.

## Figures and Tables

**Table 1:** Studies analyzing natural killer (NK) cell metabolic activity using Seahorse technology.

Species	NK Cell Metabolic Activity		Reference
	Increased by	Decreased by	
Murine NK cells	IL-2, IL-2/12, poly(I:C)	Rapamycin	[14]
	IL-15, poly(I:C)		[12]
	IL-15, IL-15+αTGF-β	Rapamycin, TGF-β	[16]
	IL-15		[13]
Human NK Cells	IL-2, IL-12/15	Rapamycin	[15]
	IL-2, IL-15	Torin	[17]
